# Bucal Secretions

**Published:** 1860-09

**Authors:** J. Taft


### Original Essays and Communications.

### THE BUCCAL SECRETIONS.

Read before the American Dental Association, at Washington, D. C., August, 1860,

BY J. TAFT.

THESE natural products of the animal economy have received some attention and consideration by physiologists and pathologists all along through medical investigation, from the beginning to the present time.

In regard to the value and importance of these secretions, there has been some diversity of opinion, or rather in regard to their functions, though they have usually been regarded as playing a very important part in the incipient stages of digestion.

The insalivation of the food, at the time of mastication, *preparatory* to deglution and digestion, have been considered the chief functions of the saliva. While these may be considered the prominent offices of this secretion, there are others accomplished by it of no insignificant character; such for instance as aiding in the enunciation of language: without the moistening, lubricating influence of the saliva, how imperfectly, if at all, would language be uttered.

Again, this product could not be retained in the blood with impunity. It is necessary, for the health of the blood, that every secretion that nature designed should be withdrawn, be duly removed by its appropriate apparatus.

Within the scope of this paper we intend chiefly to consider the buccal secretions, with reference to the influence upon the teeth, both in a state of health and disease.

It seems scarcely necessary here to enter into a minute consideration of these secretions in a state of health; since this has been so often and so elaborately done already. A few remarks here in regard to the nature, composition and elaboration of the saliva, will, however, not be out of place.

The product usually found in the mouth is denominated mixed saliva, it consists of the secretions elaborated by the buccal mucuous membrane, as well as by the various salivary glands.

The saliva, as ordinarily found in the human mouth, is an opalescent, or faintly bluish-white fluid; it is viscid, tenacious, inodorous, and tasteless. After being at rest for a time, it deposits a grayish-white sediment; this consists principally of epithelium scales; these are not seen in the fresh saliva, but soon deposit themselves when the fluid is at rest, and exposed to the air; mucous corpustles also exist to some extent in the saliva.

Unmixed Saliva. The products of the different salivary glands exhibit some variety in composition. The saliva of the same person under different physiological conditions, will be different in some respects, and in none more than in specific gravity. Various circumstances will modify it in this particular; such for instance as the amount and character of the food.

Wright has demonstrated that human saliva is more dense after food has been taken than when fasting.

The saliva of a healthy man who had lived for a week on a mixed diet varied in density from 1.0079 to 1.0085, while after a purely animal diet for an equal time, it varied from 1.0098 to 1.0176; and after a purely vegetable diet from 1.0039 to 1.0047.

He also remarks that moral emotions, (rather mental emotions) atmospheric changes, light, sound, etc., exert an influence on the density of the saliva. In two hundred cases he found the specific gravity of the saliva to range between 1.0069 and 1.0089.

Pure saliva should be obtained from the ducts of the salivary glands, in order that it may have no admixture of mucus, nor any foreign substance; it should also be obtained without the aid of irritants or stimulents. "A very simple and ready method of collecting a large quantity of saliva in a short time, is by exciting a strong pressure under the chin, and at the same time titilating the palate with a feather: a feeling of strangulation immediately ensues, during which the saliva is rapidly ejected from the mouth."

The glands that elaborate the saliva are the following, viz: the parotid, the submaxillary and sublingual. The general features of the products of these different glands are very similar; though some have affirmed that a marked difference is found in many cases. My own experiments have shown but slight difference in the saliva from the different glands.

Pure saliva is perfectly limpid and colorless, without odor or taste, and incapable of being drawn out into threads, and of a distinctly alkaline reaction.

Prolonged hunger or the use of indigestible or stimulating food causes the secretion of concentrated saliva. During fasting it approximates, and in prolonged abstinence attains an acid reaction. It is definitely alkaline during and immediately after meals. Its character is quite variable under different pathological conditions, but of this hereafter.

The saliva always possesses the following ingredients:—

*First*—Potash, soda, and lime, combined with organic matter.

*Second*—An extractive matter soluble in alcohol, precipitable by tannic acid.

*Third*—Sulpho-cyanide of potassium.

*Fourth*—Potash salt.

*Fifth*—Epithelium and mucuous corpustles; these are very slight in pure saliva.

*Sixth*—Chlorides of sodium and potassium.

*Seventh*—Traces of phosphates.

There have been many experiments and analyses made upon the saliva, almost every one of which differs in some slight degree from all the others: These differences, however, are not greater than should be expected, owing to the different specimens operated upon, and the difference in the modes of conducting the experiments. Notwithstanding the discrepances that have been exhibited by these different experiments, yet the leading features and characteristics have been manifestly the same, and fully established.

It is difficult to estimate the amount of saliva secreted in a given time, there are so many circumstances that modify the quantity, that it is difficult to arrive at any thing very definite in this respect.

It is regulated in quantity by the condition of the patient, either constitutional or local, or both. When there is no abnormal condition it will be subject to fluctuations, dependant upon the character of the food employed, and the manner of using that food. Dry food calls forth more saliva than moist, and hard far more than soft; substances of a pungent or irritating nature more than mild pleasant substances. The act of mastication is the most common exciting cause of the secretion of saliva.

The movement of the mouth in speaking and singing excites a flow, unless the speaker is in a high state of mental excitement; then the secretion may be arrested altogether, as is often experienced by timid speakers.

The flow of saliva continues for a brief period after the use of food. Food mixed with water requires very little saliva.

Colin, in his observations on the secretion of saliva, in the solidungulus animals, remarks that the secretions of the two parotids alternate, the parotid of the side on which mastication is going on secretes at least one third more than that of the other side; and when mastication is changed to the other side the corresponding parotid goes vigorously to work: the action of the first partially ceases.

The same rule operates in the secretion of the human saliva, though probably not to quite the same extent.

The parotid glands yield about two thirds of the whole amount of the saliva elaborated; the sub-maxillary about one-twentieth and the sublingual the remainder. The amount of saliva is governed by circumstances. During sleep there is little or no saliva secreted. The sight and odor of food will usually excite a flow of saliva, particularly if there is hunger. Nausea will generally excite a free flow of the buccal fluids; at such a time the mucuous glands become especially active.

The various mental conditions operate variously upon the salivary glands: Fear diminishes the secretion very much; fright checks it entirely; anger also diminishes it; while good humor and mirth increases the secretion. Erotic emotions increase the secretion of the mucuous considerably, and that of the saliva to some extent. Grief augments the secretion of both the saliva and mucus.

Fluctuations in the quantity of water in the blood has but little influence upon the amount of saliva elaborated in a given period. Neither does it change the relative proportion of the solid constituents of the saliva.

THE OBJECTS OF THE SALIVA.—Lehmann remarks, that the office of the saliva may be regarded as three fold, viz: mechanical, chemical and dinamical.

Many kinds of food have but little moisture, and require to be moistened with the saliva in order that more perfect mastication may be accomplished, and especially that the act of deglution may be accomplished with facility; it lubricates the bolus and makes it manageable in the mouth. Perfectly dry food in the mouth is unmanageable. Without the moistening and lubrication of the food in the mouth, the mucous membrane would be irritated, and ultimately highly diseased by friction; this would result to the mucous membrane of the throat and esophagus, as well as to that of the mouth.

The food is prepared by insalivation for being most perfectly acted upon by the digestive fluids. *The chemical* action of the saliva upon the food is a question upon which there is much diversity of opinion; some maintaining that it serves only to moisten the food, and at that point its influence ceases; others contend that it exercises a concurrent influence with the gastric fluids throughout the process of digestion. There are some points, however, that are well established. By insalivation the food is much better prepared for being acted upon by the gastric fluid than if it was not moistened at all, or than if moistened simply with water. Again, starch is converted into sugar by the action of the mixed saliva. This is probably accomplished by the mucus. Experiments have been introduced that lead us to conclude that the unmixed saliva will not produce that result.

Lehmann remarks, "it can, therefore, no longer be doubted that the saliva in a normal condition, when it is mixed with the food, possesses the property of converting starch into sugar."

Wright considers that one of the more prominent functions of the saliva is that of stimulating the stomach, and thus promoting the digestive process. This rests, however, quite as much upon hypothesis as demonstration.

Pathological Conditions.—The diseased and unnatural conditions of the buccal secretions have not received that attention and investigation which we think the subject demands; this arises from the supposition that the subject is one of little importance; and from the difficulties attending such investigation.

The saliva is subject to many and great changes, by disease. The saliva proper may be changed from its natural to a definitely acid reaction; or it may be changed to more than its alkaline condition. This latter is exhibited when salivary calculus is rapidly deposited upon the teeth.

Any change of the mucus is usually to an increased acidity. When this condition is produced to such an extent that it is not neutralized by the saliva, as is generally the case when green tartar is deposited upon the teeth, it then becomes a source of mischief.

Probably in no cases except those of an extreme character is there any direct injury upon the teeth by these changes of the saliva; but that the decomposition of foreign substances in the mouth is greatly facilitated by these changes, there can be no doubt.

The extent of the injury resulting from vitiated secretions will be exceedingly various, depending upon a great variety of modifying circumstances.

The saliva becomes changed to an abnormal or diseased condition in two ways. First, by a vitiated state of the blood, and second, by a diseased condition of the glands; the latter is often complicated with the former. The saliva is more frequently affected by a deteriorated state of the blood; but the mucus by an unhealthy condition of the mucous follicles. These being situated in the mucous membrane near the surface, are more exposed than the salivary glands to the influence of local irritating causes.

The salivary glands are often required to perform more than the natural amount of labor, sometimes from local stimulus, as in the use of tobacco, or any similar agent; and from the constant use of the jaws in mastication, and from general constitutional causes, as in ptyalism, and various vitiated conditions of the blood.

When any organ is over-tasked, its production is necessarily deteriorated. A general inflammatory condition of the system produces an acid state of the saliva. Rheumatic affections, if general, produce the same result.

In febrile diseases the secretion of the saliva is very much diminished and sometimes checked altogether; then, the secretion of mucus being kept up, the teeth suffer injury, for the mucus, at such times, is always in a vitiated condition when secreted, and changes rapidly to a worse condition while in the mouth. The water evaporates and leaves a thick tenacions sordes covering the teeth, and lining the mucous membrane of the mouth. During this state of things, the teeth, without great care, are injured extensively and rapidly. The utmost care should be exercised to keep the teeth clean during a time of sickness, and especially when this peculiar condition exists. Much of the injury attributed to medical agents is dependent upon just the state of things to which reference has been made.

This vitiated, thickened, decomposing mucus will act directly and with great rapidity upon the teeth. It also facilitates the decomposition of the foreign substances with which it may come in contact, or commingle, and thus indirectly operate an injury upon the teeth.

A peculiarly vitiated state of the saliva is often found in connection with a lymphatic temperament, especially where there is a strumous diathesis. The saliva is viscid, somewhat thickened, is easily drawn out into shreds or strings, it does not readily mix with the atmosphere, does not become frothy. Usually when there is this state of the saliva, the teeth decay very rapidly, and are very sensitive, especially if the constitution of the teeth is good or medium ; but if they are poorly organized, the vitality may be destroyed in advance of the decay. Saliva in this condition operates rapidly upon the teeth, both directly and indirectly. All the care that can be bestowed upon the teeth in respect to cleanliness will not arrest caries nor change its character while the buccal secretions remain in the condition just described. The mucous membrane of the mouth is always in a more or less irritable condition in these cases, and this will be kept up during this state of the saliva, and probably by its influence chiefly.

In order to remedy the evil results from this condition of the saliva, it must be restored to health ; this can only be done by constitutional treatment, for it doubtless depends upon a depraved state of the blood. The secreting organs are, doubtless, always more or less affected; but when the original cause of the difficulty is removed, the secreting apparatus will assume a normal condition and a healthy action.

There is another depraved condition of the saliva not so marked in character nor injurious in its effects as that just referred to. It is characterized by a semi-oily condition; it is free from that tenacity and stringiness exhibited by some other depraved conditions. It insinuates itself into every interstice where it can at all approach; it will moisten the surfaces of the teeth when the utmost care is taken to protect them; the dentist will thus often find himself exceedingly annoyed by saliva of this character. Where it exists, it is almost impossible to keep an operation dry. This peculiarity is derived from the mucus, which in such instances is elaborated very freely.

The pathological changes and conditions to which the buccal secretions are subject is a matter upon which but little has been written, and less investigation made, especially with reference to their influence upon the teeth. It is a field in which investigation is somewhat difficult, yet there are no insurmountable difficulties in the way; it may be as thoroughly explored as some that have received much attention. It is an exceedingly important subject to the dentist and his patient; one that we all have neglected too long. Now in the future let us give this subject the attention its importance demands.

